# Multimodal Method for Differentiating Various Clinical Forms of Basal Cell Carcinoma and Benign Neoplasms In Vivo

**DOI:** 10.3390/diagnostics14020202

**Published:** 2024-01-17

**Authors:** Yuriy I. Surkov, Isabella A. Serebryakova, Yana K. Kuzinova, Olga M. Konopatskova, Dmitriy V. Safronov, Sergey V. Kapralov, Elina A. Genina, Valery V. Tuchin

**Affiliations:** 1 Institution of Physics, Saratov State University, 410012 Saratov, Russia; s.izabell2014@gmail.com (I.A.S.); eagenina@yandex.ru (E.A.G.); 2 Laboratory of Laser Molecular Imaging and Machine Learning, Tomsk State University, 634050 Tomsk, Russia; 3 Laboratory of Biomedical Photoacoustic, Saratov State University, 410012 Saratov, Russia; o.konopatskova@mail.ru; 4 Department of Faculty Surgery and Oncology, Saratov State Medical University, 410012 Saratov, Russia; yan.prokhorova@gmail.com (Y.K.K.); dr.safronof@yandex.ru (D.V.S.); sergejkapralov@yandex.ru (S.V.K.); 5 Institute of Precision Mechanics and Control, FRC “Saratov Scientific Centre of the Russian Academy of Sciences”, 410028 Saratov, Russia

**Keywords:** basal cell carcinoma, benign neoplasm, diffuse reflectance spectroscopy, optical coherence tomography, high-frequency ultrasound, texture image analysis, multimodal diagnostic method, machine learning

## Abstract

Correct classification of skin lesions is a key step in skin cancer screening, which requires high accuracy and interpretability. This paper proposes a multimodal method for differentiating various clinical forms of basal cell carcinoma and benign neoplasms that includes machine learning. This study was conducted on 37 neoplasms, including benign neoplasms and five different clinical forms of basal cell carcinoma. The proposed multimodal screening method combines diffuse reflectance spectroscopy, optical coherence tomography and high-frequency ultrasound. Using diffuse reflectance spectroscopy, the coefficients of melanin pigmentation, erythema, hemoglobin content, and the slope coefficient of diffuse reflectance spectroscopy in the wavelength range 650–800 nm were determined. Statistical texture analysis of optical coherence tomography images was used to calculate first- and second-order statistical parameters. The analysis of ultrasound images assessed the shape of the tumor according to parameters such as area, perimeter, roundness and other characteristics. Based on the calculated parameters, a machine learning algorithm was developed to differentiate the various clinical forms of basal cell carcinoma. The proposed algorithm for classifying various forms of basal cell carcinoma and benign neoplasms provided a sensitivity of 70.6 ± 17.3%, specificity of 95.9 ± 2.5%, precision of 72.6 ± 14.2%, F_1_ score of 71.5 ± 15.6% and mean intersection over union of 57.6 ± 20.1%. Moreover, for differentiating basal cell carcinoma and benign neoplasms without taking into account the clinical form, the method achieved a sensitivity of 89.1 ± 8.0%, specificity of 95.1 ± 0.7%, F_1_ score of 89.3 ± 3.4% and mean intersection over union of 82.6 ± 10.8%.

## 1. Introduction

Basal cell carcinoma is one of the most common skin tumors worldwide [1]. The risk of developing basal cell carcinoma (BCC) increases with factors such as genetic predisposition, increased life expectancy, low melanin content in the skin, frequent and prolonged exposure to ultraviolet radiation on exposed areas of the body and other environmental influences [2,3,4,5]. According to [6], the probability of receiving a diagnosis of BCC in Caucasians over 60 years of age is estimated at 11%. However, the average lifetime risk of developing BCC in people with white skin is approximately 30% [7]. Over the next 10 years, the incidence of BCC is expected to increase by 30% (men) and 25% (women) [7].

Estimating the prevalence of BCC is challenging because not all cases of the disease are reported by official medical statistics [8]. However, in recent years, there has been an increase in the rate of growth in the number of cases of this disease, which leads to increased attention toward the diagnosis of this disease [9]. BCC can have various clinical manifestations (forms), such as superficial, nodular, morpheaform, micronodular and infiltrative, as well as pigmented, mixed, etc. [6,10]. The malignant potential of BCC lies in its locally invasive and destructive nature, which can lead to functional and cosmetic complications [11]. Current guidelines for the treatment of BCC suggest different approaches depending on the clinical form of BCC [6,12]. Therefore, the correct differentiation of clinical forms of BCC is crucial for choosing the most appropriate therapy. However, the choice of treatment tactics for patients with BCC should be made individually, taking into account not only the clinical form but also the extent of the tumor process, its localization and other prognostic factors.

At the moment, biopsy and histopathology are the main methods for diagnosing and establishing the clinical form of BCC [12,13]. Based on the results of the biopsy, a decision is made on the appropriate treatment method. In practice, various biopsy techniques may be used, including excisional, incisional, shave and punch biopsies. For example, the diagnostic accuracy of punch biopsy compared with post-surgical histopathology ranges from 61 to 82 percent [13,14].

Despite the differences in clinical features, all subtypes of basal cell carcinoma have a common histopathological feature: the presence of aggregates of basaloid keratinocytes surrounded by stromal tissue and, as a rule, connected to the epidermis [13,15]. Externally, basaloid cells resemble normal basal keratinocytes of the epidermis. They have a large and relatively uniform nucleus and scanty cytoplasm. The nodular subtype of BCC is characterized by large accumulations of basaloid cells in the papillary or reticular dermis, accompanied by stromal retraction around the tumor and peripheral palisading. Phenomena of ulceration and cystic spaces are observed, which can form inside large tumor islands due to necrosis [13,15]. In superficial BCC, basaloid cells develop along an axis parallel to the surface of the epidermis, without penetrating deeper than the papillary dermis. There may be a narrow retraction of the palisading arrangement of basaloid cells from the underlying stroma. These tumors can be characterized as multifocal if multiple distant foci of cell proliferation are present; however, they connect with each other to form a reticular pattern, causing most superficial BCCs to not be truly multifocal [13,15]. Typical growth of morpheaform BCC is characterized by the penetration of thin bundles of basaloid cells, consisting of one to five layers, between dense bundles of collagen. The tumor is often poorly demarcated and may demonstrate invasion into the reticular dermis and even penetration into the subcutaneous fat [13,15]. Infiltrative BCC demonstrates heavy stromal fibrosis with dense collagen bundles, grows in a poorly circumscribed fashion and may show invasion of the subcutis. However, tumor cells form large nodules with irregular contours in addition to strands and cords [13,15]. Similar to nodular BCC, micronodular BCC shows round or oval tumor nests that are smaller and widely dispersed, penetrating deeper into the dermis and, in some cases, even penetrating the subcutaneous fatty tissue [13,15].

Thus, it may be useful to develop methods for a rapid non-invasive preoperative assessment of tumor extent and differentiation of clinical forms of neoplasms, allowing one to avoid intervention for diagnostic purposes, reducing both patient morbidity and financial costs associated with surgery.

There is a large number of medical imaging techniques that can be used as primary data sources to create a diagnostic method. When choosing an imaging method for a targeted clinical application, it is necessary to take into account the specificity of this method for the organs being studied. In practice, it is not possible to capture all the details with a single imaging modality that would ensure the clinical accuracy and reliability of the analysis and subsequent predicted diagnosis. Various research methods have been developed for effective diagnosis of BCC [16,17]. The main ways to detect a tumor are visual examination of the skin and dermatoscopy, which depend on the subjective opinion of the oncologist. Traditional methods for diagnosing BCC, such as biopsy, cytology and histology, have their drawbacks, such as mechanical damage to the skin surface and prolonged sample preparation. Therefore, much attention is paid to the search and development of innovative methods for diagnosing BCC, such as optical coherence tomography (OCT) [18,19,20], high-frequency ultrasound (US) [21,22,23,24] and diffuse reflectance spectroscopy (DRS) [25,26,27]. For example, Silver et al. [28,29] use vibrational OCT in conjunction with machine learning to differentiate cancerous tissues and normal skin according to the estimated stiffness coefficients of each tissue component. Since these methods are non-invasive, they can be used as tools for monitoring treatment response and the timely detection of BCC recurrence. Spectroscopy and refractometry methods in a wide spectral range make it possible to detect changes in the composition and morphology of tissues during the progression of tumors. OCT and US are the two main methods that allow for visualization of the internal structure of various forms of skin neoplasms in vivo.

While US provides good information about the depth and structure of BCC, its resolution is limited, which can sometimes lead to difficulties in differentiating BCC from other cutaneous structures and conditions [21,22,23,24]. OCT offers high-resolution imaging and can give detailed structural information of the epidermis and dermis that can be missed by US. Its ability to image skin morphology complements US by providing additional information that can help to differentiate BCC from other skin pathologies with similar sonographic appearances [18,19,20]. DRS provides functional information about the tissue, such as the concentration of hemoglobin and melanin. This can enhance the detection of BCC by identifying areas with increased vascularity and pigmentation changes that are characteristic of cancerous tissues [25,26,27].

The integration of these imaging techniques aims to leverage their synergistic capabilities to increase the overall accuracy and diagnostic confidence. By combining structural information from US and OCT with the functional data from DRS, we aim to reduce the potential for false positives and negatives, providing a more robust and reliable diagnosis of BCC. This multimodal approach could be particularly beneficial for ambiguous or challenging cases where a single modality might provide incomplete information.

Since each type of neoplasm is characterized by unique changes in skin structure, these features can be used as a diagnostic marker. In order to differentiate the clinical forms of malignant and benign neoplasms (BNs), it is useful to develop methods for texture analysis of digital images necessary to quantify changes in skin structure and eliminate diagnostic errors due to variability in the visual perception of the oncologist, which can help in making objective clinical decisions [6,12].

A multimodal approach to diagnostics is a relatively new trend that builds models and diagnostic methods that take into account information from different modalities and establish correlations between features [30,31]. The results of many scientific groups show an increase in diagnostic accuracy when moving from single-modal to multimodal methods. Additionally, multimodal approaches can allow for the absence of some attributes of any modality and still maintain a more robust method performance [30]. The development of multimodal methods provides a greater variety of assessed parameters used for medical diagnosis, leading to more reliable predictions of diagnosis and patient condition that may be unnoticed or underestimated by single-modal screening [30,32].

This paper presents the possibilities of differentiating various clinical forms of BCC and BN using texture analysis of OCT images of neoplasms, analysis of the shape of the tumor from US images and DRS in the range of 450–950 nm.

## 2. Materials and Methods

### 2.1. Object and Methods of Research

Selection criteria were the following: morphologically confirmed diagnosis of basal cell skin cancer (according to the International Classification of Skin Tumors, WHO, 2018); the age of patients is from 18 to 85 years; signed informed consent of the patient to participate in the study. Exclusion criteria were the following: morphologically confirmed diagnosis of skin melanoma; morphologically confirmed diagnosis of squamous cell carcinoma of skin; open wounds and ulcers in the diagnostic area; and patient refusal to participate in clinical trials.

Finally, the study involved 37 volunteers of both sexes with BN or BCC aged from 40 to 84 years with the second type of pigmentation according to the Fitzpatrick skin type classification scale [33]. The study was approved by the Ethical Committee of Saratov State Medical University named after V.I. Razumovsky of Ministry of Health of Russia (protocol №09 from 4 April 2023) and all investigations were performed in accordance with the ethical standards of the Declaration of Helsinki [34] or comparable ethical standards.

We studied 37 neoplasms diagnosed as BCC or BN, which were divided into 6 classes: infiltrative–ulcerative (quantity 2), pigmented (quantity 2), superficial (quantity 15), morpheaform (quantity 3) and nodular (quantity 5) clinical form of BCC, and BN (quantity 10), which included nevi, fibromas and dermatofibromas.

To visualize the skin surface with a lesion, a Firefly DE 300 polarizing dermatoscope (Firefly, Luneburg, Germany) with 10× magnification and a resolution of 1920 by 1080 pixels was used. High-frequency US was performed using a DUB SkinScanner device (Tpm Taberna pro Medicum, Luneburg, Germany) in B-scan mode with two probes operating at central frequencies of 33 and 75 MHz. Scanning depth was up to 5 mm and axial resolution was 48 and 21 μm, respectively. OCT scans were obtained using spectral OCT GAN930V2-BU (Thorlabs, Newton, NJ, USA), operating at a central wavelength of 930 nm with an axial and lateral resolution of 5.34 and 7.32 μm (in air), respectively, and a scanning depth of 2.7 mm. The diffuse reflectance (DR) spectrum was recorded using a USB4000-UV-VIS spectrometer (Ocean Optics, Peabody, MA, USA) and a QR400-7-VIS-NIR fiber-optic probe (Ocean Optics, Peabody, MA, USA) in the range of 450–950 nm.

**Figure 1:**
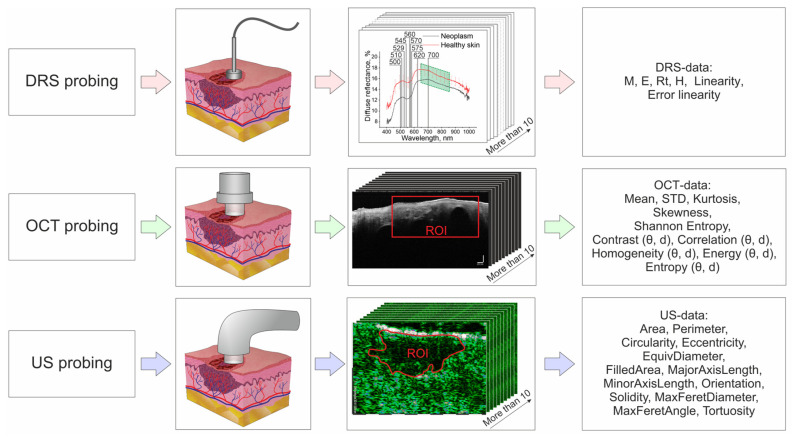
Scheme of DRS, OCT and US probing of a tumor and extraction of parameters of each modality presented on the example of the data of the nodular clinical form of BCC.

Figure 1 shows a block diagram and order of DRS, OCT and ultrasound probing of a tumor and extraction of parameters of each modality.

### 2.2. Analysis of DR Spectra

To take into account the individual spectral characteristics of the volunteer’s skin, for each neoplasm, DR spectra were recorded from nearby or symmetrically located lesions of healthy skin areas.

In order to analyze the spectra of the DR coefficient, 10 spectra were selected for each neoplasm (see Figure 1).

The DR at wavelength λ (R_λ_) was calculated as the ratio of the intensity of radiation reflected from the sample to the intensity of radiation reflected from a reference sample with reflectivity close to 100% in the wavelength range from 450 to 950 nm.

Based on the spectra of the DR, the coefficients of melanin pigmentation (M), erythema (E), hemoglobin content (H), the slope coefficient of the DR spectrum (Linearity) in the wavelength range 650–800 nm and the coefficient characterizing the deviation from linearity approximation (Error_linearity) were calculated. To exclude individual spectral features of the skin of volunteers, each coefficient calculated for a neoplasm was normalized to the average coefficient calculated for healthy skin.

The melanin pigmentation index is used to quantify the melanin content of a sample. The melanin pigmentation index M of human skin was determined using the formula [35]:

(1) $$M=100\left({\mathrm{OD}}_{620}-{\mathrm{OD}}_{700}\right),$$

where ${\mathrm{OD}}_{\lambda }=-\mathrm{lg}R_{\lambda }$ is the effective optical density at wavelengths λ.

The method for determining the severity of erythema of human skin is based on measuring the spectral dependence of the diffuse reflectance coefficient of the skin in the range of 510–610 nm. The erythema index E was calculated using the formula [36]:

(2) $$E=100\;\left[{\mathrm{OD}}_{560}+1.5\;\left({\mathrm{OD}}_{545}+{\mathrm{OD}}_{575}\right)-2\;\left({\mathrm{OD}}_{510}+{\mathrm{OD}}_{610}\right)\right].$$

To estimate the hemoglobin content H, the following expression was used [36]:

(3) $$H=\frac{{\mathrm{OD}}_{545}-{\mathrm{OD}}_{529}}{16}-\frac{{\mathrm{OD}}_{570}-{\mathrm{OD}}_{545}}{25}.$$

Linearity and Error_linearity coefficients in the wavelength range 650–800 nm were calculated using standard functions of MATLAB version R2019a (The MathWorks, Natick, MA, USA).

In addition to the described coefficients, the “malignancy” coefficient (Rt) was calculated from the spectra of the DR [26] using the formula:

(4) $$\mathrm{Rt}=\frac{R_{\mathrm{healthy}\;500}\;R_{\mathrm{neoplasm}\;700}}{R_{\mathrm{healthy}\;700}\;R_{\mathrm{neoplasm}\;500}},$$

where $R_{\mathrm{healthy}\;500},{\;R}_{\mathrm{healthy}\;700}$$,{\;R}_{\mathrm{neoplasm}\;500},{\;R}_{\mathrm{neoplasm}\;700\;}$are the coefficient DRS of visually healthy skin and neoplasm at wavelengths 500 and 700 nm, respectively.

### 2.3. Texture Analysis of OCT-Images

Texture analysis is the analysis of changes in pixel brightness in a small area of an image. Textural characteristics of an image quantify properties such as homogeneity, coarseness and contrast. In the context of OCT, texture analysis allows for quantification of tissue heterogeneity in a selected area of a structural image. This analysis is widely used in processing various types of images [37] despite the fact that, currently, the concept of texture is not well defined and texture parameters are often interpreted differently. Haralik noted [38] that there is no formal approach to describing and defining texture, and texture extraction methods are usually developed on a case-by-case basis. At least three different approaches to texture analysis can be found [39]: statistical, structural and stochastic.

In statistical texture analysis, texture characteristics are calculated based on the statistical distribution of image pixel intensities within a region of interest (ROI). Statistical methods are divided into statistical methods of first order (first-order statistics, FOS), second order (second-order statistics, SOS) or gray-level co-occurrence matrix (GLCM), and higher-order statistics [20,40,41,42,43]. FOS parameters such as mean, standard deviation, skewness and kurtosis are directly related to the grayscale distribution of pixel intensities and ignore inter-pixel correlations. SOS, on the contrary, depends on the spatial arrangement of pixel intensities in the ROI. SOS parameters include energy, homogeneity or inverse difference moment, contrast, correlation and entropy.

GLCMs describe the SOS of images. In this case, the statistics depend on the spatial arrangement of gray levels in the region of interest and provide texture information for that region. This method is based on estimating joint second-order conditional probability density functions P_d_,_θ_(i, j). Each P_d_,_θ_(i, j) represents the probability of transition from gray level i to gray level j in a given direction θ for a given interpixel spacing d [38,41].

The energy parameter takes the smallest value when all probability density functions P_d,θ_(i, j) are equal and there are no dominant gray levels; that is, when most gray levels are equally probable [38,41].

The contrast parameter is very sensitive to large differences occurring within the GLCM. Highly contrasting areas have high contrast, while more uniform areas have low contrast [38,41].

Correlation quantifies the gray level dependence between two pixels separated by a distance d. Low correlation means that the gray levels tend to be independent of each other, meaning that there is no regular structure in the image. However, if the correlation is high, there is a possibility that one or more patterns are repeated within the computational window [38,41].

Entropy measures the lack of spatial organization within a computational window. Entropy is high when all P_d,θ_(i, j) are unequal, corresponding to a rough texture, and low when the texture is more uniform or smoother [38,41].

The homogeneity parameter quantifies local similarity within a computational window. It is expected to be higher for GLCMs with elements concentrated near the diagonal. These GLCMs correspond to textures of organized and poorly contrasted elements, with only a few gray levels at equal distance d from each other. This parameter quantifies the degree of homogeneity in the ROI [38,41].

**Figure 2:**
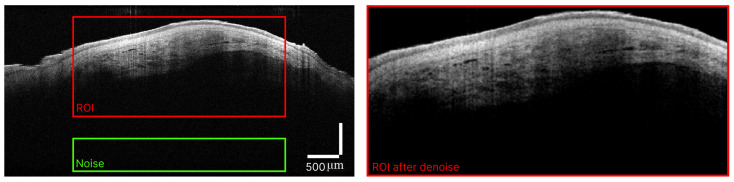
Typical raw OCT scan of morpheaform BCC with ROI and area outside the structural image marked and ROI after denoising.

For texture analysis, 370 OCT images were selected that did not contain artifacts, such as motion artifacts that can lead to blurring of the structural image, specular reflection artifacts and shadow effects in which the reflection of light from dense structures can cause the underlying tissue to be visualized darker in comparison with neighboring identical biological tissues. Researchers with experience in interpreting OCT images and having publications on the topic of OCT studies of neoplasms, together with experienced oncologists, identified the ROIs for each OCT B-scan. They contained images of the internal structure of the neoplasm for subsequent calculation of FOS and Haralick parameters. The ROIs were at least 534 μm deep and at least 400 μm wide. For each tumor, 10 OCT images with one region of interest were selected, and each B-scan was recorded in different areas of the tumor (see Figure 1 and Figure 2). ROI selection was necessary because some neoplasms were smaller than the width of the OCT probing window or involved a relatively healthy area of skin. In addition, some neoplasms were localized in anatomically uneven areas, such as the nose, and difficult to study using the OCT method, while only part of the image was in the focus of the OCT system.

In order to reduce the influence of noise outside the structural image of the biological tissue, a threshold filter was used. The intensity of each pixel was compared with a threshold value; if the intensity was less than or equal to the threshold, the intensity of this pixel was taken as equal to 0. To determine the threshold value on each OCT scan, a rectangular area measuring approximately 500 by 2000 μm was selected below the image of the biological tissue, according to which the mean and standard deviation of pixel intensity were calculated. The threshold value was the sum of the mean and standard deviation. To reduce the influence of speckle noise on B-scans, an erosion filter with a window width of 3 pixels and a two-dimensional smoothing filter with a Gaussian kernel with a specified standard deviation of 1.5 were successively used.

Figure 2 shows the original OCT scan with the ROI marked for calculating the FOS and Haralick parameters and the region used to estimate the threshold signal before and after threshold filtering.

To calculate the Haralick parameters, 4 GLCMs were first calculated in the region of interest. The GLCM matrices were calculated for four orientations: horizontal, vertical and two diagonals (directions defined by four angles: 0°, 45°, 90° and 135°). The interpixel spacing was 1, 5 and 9 pixels. In this way, 12 GLCMs were obtained for 4 directions and 3 interpixel intervals for each ROI from the 8-bit OCT image. For each GLCM, five Haralick characteristics (SOS parameters) were calculated. Four FOS parameters were calculated from the histogram of gray level distribution in the ROI. Additionally, Shannon entropy was calculated for each ROI from the original B-scan.

Using MATLAB, we developed software for automatic calculation of the parameters. Thus, a total of 65 characteristic parameters were obtained for each ROI. Pixels with intensity equal to zero, associated with the background, were not taken into account when estimating the FOS and Haralick parameters.

### 2.4. US Image Analysis

To analyze US data, 10 US images without artifacts and having the most contrasting boundaries of the tumor with conditionally healthy skin were selected for each neoplasm (see Figure 1).

To reduce the influence of human subjectivity, two authors of this work, independently of each other, identified ROI along the visible boundaries of a tumor with healthy skin. To analyze the shape of the tumor, such areas were used, which represented the intersection of the ROIs identified by both authors.

**Figure 3:**
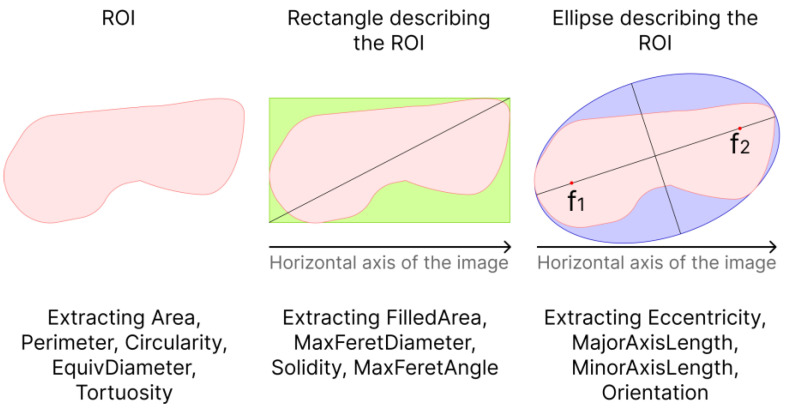
Scheme for analyzing the shape of a neoplasm, where f_1_ and f_2_ are the ellipse focus.

When analyzing the tumor shape, the following parameters were calculated: area (Area), perimeter (Perimeter), roundness (Circularity) of the ROI, equivalent diameter of a circle (EquivDiameter), ratio of perimeter to area (Tortuosity), eccentricity of the ellipse (Eccentricity), length of the major axis of the ellipse (MajorAxisLength) and the length of the minor axis of the ellipse (MinorAxisLength) describing the ROI, the magnitude of the angle between the major axis of the ellipse and the horizontal axis of the image (Orientation), the area of the rectangle (FilledArea), the maximum diameter of the rectangle (MaxFeretDiameter) describing the ROI, the ratio of the area of the ROI to the area rectangle (Solidity) and the angle between the maximum diameter of the rectangle and the horizontal axis of the image (MaxFeretAngle) (see Figure 3).

The area was calculated as the sum of pixels in the ROI. The perimeter was calculated as the sum of the distances between each adjacent pair of pixels around the border of the ROI.

Circularity was calculated as:

(5) $$\mathrm{Circularity}=\left(4\cdot \mathrm{Area}\cdot \pi \right)/{\mathrm{Perimeter}}^{2}.$$

The eccentricity of the ellipse describing the ROI was calculated as the ratio of the distance between the foci of the ellipse and its major principal axis.

The equivalent diameter of a circle having the same area as the ROI was calculated as:

(6) $${\mathrm{EquivDiameter}}^{2}=4\cdot \mathrm{Area}/\pi .$$

Thus, for each ultrasound image, 13 parameters were calculated, depending on the shape and size of the neoplasm.

### 2.5. Machine Learning

After calculating, 6 parameters related to the DRS data, 65 parameters related to the OCT data and 13 parameters related to the high-frequency US data for each measurement were compared with the diagnosis of the oncologist and compiled into a single table, where the columns corresponded to the parameter and rows to the measurements (observations). Due to the spatial inconsistency of measurements of DR spectra, OCT scans and high-frequency US images, each observation was randomly assigned a row of 3 groups of parameters extracted from DRS, OCT and US within the same tumor so as to avoid duplication. For example, the 1st observation of one of the tumors was associated with the 2nd row from the group of parameters extracted from the DR spectra, the 1st row from the group of parameters extracted from OCT data and the 5th row from the group of parameters extracted from high-frequency ultrasound data.

**Figure 4:**
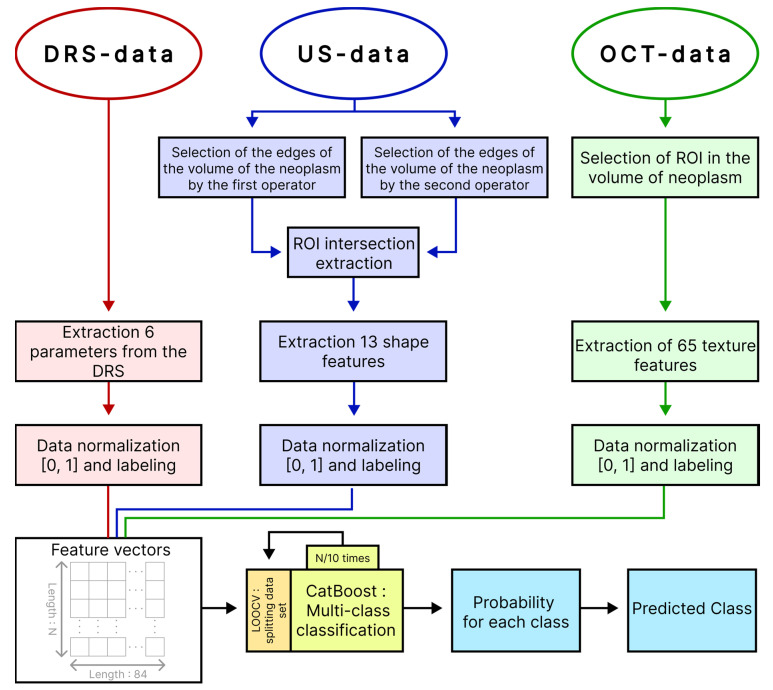
Block diagram of the proposed algorithm for multiclass classification of neoplasms.

Based on the calculated parameters, we built a multi-class classifier of BN and various clinical forms of BCC that is based on the machine learning method using gradient boosting decision tree, implemented in the CatBoost library. To build the classifier and evaluate its quality, we used leave-one-out cross-validation (LOOCV), in which all observations of one tumor were removed from the training set to avoid biasing the accuracy estimate due to potential correlations between data from the same tumor. At each iteration, the classifier was trained on feature vectors from all tumors except one and tested on the remaining feature vector of the tumor. Thus, all data participated in training and testing, while data recorded from one tumor were not simultaneously included in the training and testing sets. The built-in loss function “MultiClass” was used as a learning metric. In this study, CatBoost hyperparameters were tuned through trial and error. Classifier training stopped when the built-in overfitting detector was triggered. Before analysis, all parameter groups were scaled to range from 0 to 1. To reduce imbalance in the sample, we assigned more weight to observations from the smaller class and less weight to observations from the larger class. At the output, the model produced a vector of probabilities of the test dataset belonging to each of the diagnoses. The block diagram of the proposed approach to multi-class classification of neoplasms is presented in Figure 4.

To assess the quality of the developed algorithm for multiclass classification, the following parameters were used: sensitivity, specificity, accuracy, F_1_ and mean intersection over union (mIoU).

The main objective of the classification is to determine the type and clinical form of skin neoplasm. Sensitivity and specificity are commonly used to numerically evaluate the quality of a classification algorithm. Sensitivity or completeness (Sens) is the proportion of correctly found positive objects among all objects of the positive class:

(7) $$\mathrm{Sens}=\frac{\mathrm{tp}}{\mathrm{tp}+\sum \mathrm{fn}},\;$$

where tp is the proportion of true positives and fn is the proportion of false negative predictions.

Specificity (Spec) measures the proportion of actual negative results that are correctly identified as such:

(8) $$\mathrm{Spec}=\frac{\sum \mathrm{tn}}{\sum \mathrm{tn}+\sum \mathrm{fp}},\;$$

where tn is the proportion of true negatives and fp is the proportion of false positives.

Metrics such as precision can be used as evaluation metrics for a multiclass classification algorithm. Precision (Prec) is the proportion of true predictions within all positive predictions of the algorithm:

(9) $$\mathrm{Prec}=\frac{\mathrm{tp}}{\mathrm{tp}+\sum \mathrm{fp}}.$$

It is also convenient to use the F_1_-measure (or F-score), which combines precision and specificity with an equally weighted average:

(10) $$F_{1}=\frac{2\times Prec\times Sens}{Prec+Sens},\;$$

Also, as a measure of the quality of the proposed method for each class of neoplasm, the intersection by union (IoU) metric was used:

(11) $$\mathrm{IoU}=\frac{\mathrm{tp}}{\mathrm{tp}+\sum \mathrm{fn}+\sum \mathrm{fp}}.$$

To adequately assess the quality of a multiclass classification algorithm when assessing precision, specificity, F_1_ and IoU in the case of class imbalance, it is necessary to calculate the precision, specificity and IoU for each class separately and then average it.

The importance or contribution of features to the model prediction was assessed using the built-in function in the CatBoost library (get_feature_importance(test_feature_vectors)). The importance of a feature was assessed by its influence on the loss function that was used for training. For the second method of assessing the influence of a feature on prediction, the functionality of the SHAP library was used. The SHAP library calculates Shapley values [43] to evaluate the importance of a parameter. Shapley values quantify the contribution of each feature to the final prediction for arbitrary machine learning models.

## 3. Results

**Figure 5:**
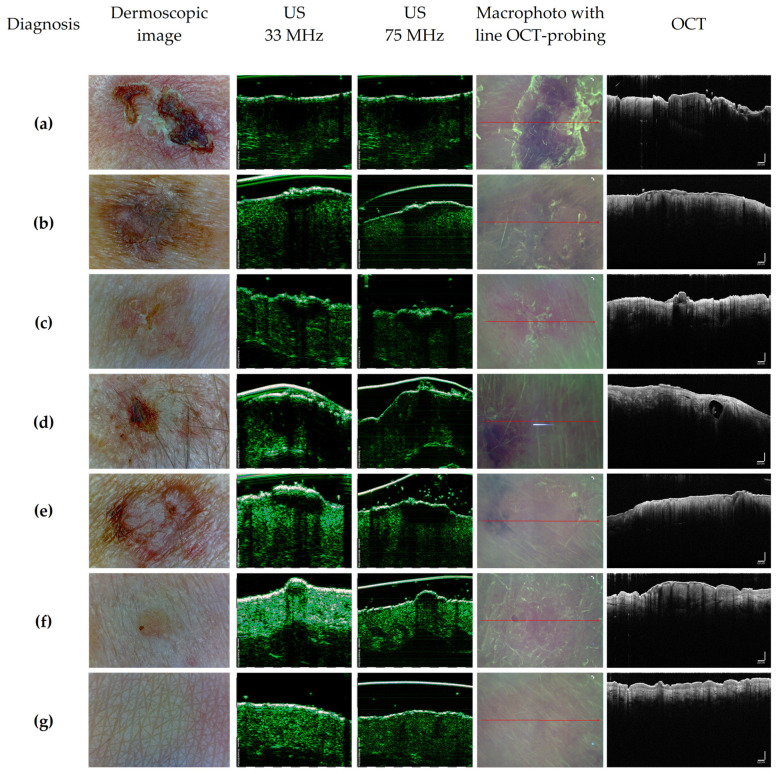
Typical dermoscopic images, ultrasound images at a central frequency of 33 MHz and 75 MHz, macro images with the plane and direction of OCT scanning marked with a red arrow and OCT scans of skin neoplasms of volunteers with a diagnosis of BCC infiltrative–ulcerative (**a**), pigmented (**b**), superficial (**c**), morpheaform (**d**), nodular (**e**), benign neoplasm (**f**) and visually healthy skin (**g**). The height and width of ultrasound images were 3.2 and 13 mm, and those of OCT images were 2.7 and 6 mm.

Figure 5 shows typical dermoscopic, US and OCT images of BN and various clinical forms of BCC. As shown in Figure 5, in ultrasound images, the tumor volume is visualized as a hypoechoic area, while, in visually healthy skin, there is no hypoechoic area. In OCT images of healthy skin, the boundary between the epidermis and dermis is clearly defined, while, for all neoplasms, this boundary is absent or has less contrast. Also, in ultrasound images, the internal structure of the tumor is not visualized, while, in OCT images, the structure of the tumor is visualized.

**Figure 6:**
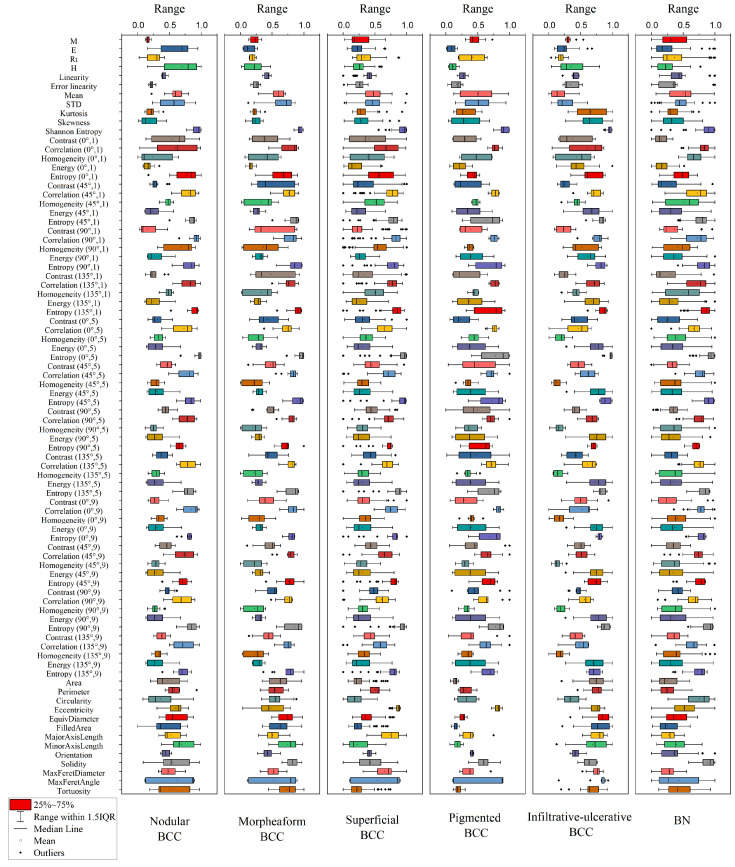
Distribution of parameter values extracted from DRS, OCT and US for various diagnoses. The angle and interpixel intervals used in the calculation of the gray-level co-occurrence matrix for the ROI of OCT images are indicated in parentheses.

Figure 6 shows the distributions of parameter values for each diagnosis extracted from this DRS, OCT and US. To simplify the classification model, i.e., to reduce the dimension of the coefficient space, parameters correlating with each other can be taken into account using principal component analysis. As can be seen in Figure 6, the melanin pigmentation coefficient (M) has the highest average value for the BN groups and the pigmented form of BCC. The erythema index (E) and hemoglobin index (H) have the highest average values for the nodular form of BCC while, for the superficial form of BCC, these parameters have low values. The circularity parameter has a larger value for BN and a smaller value for the superficial clinical form of BCC.

**Figure 7:**
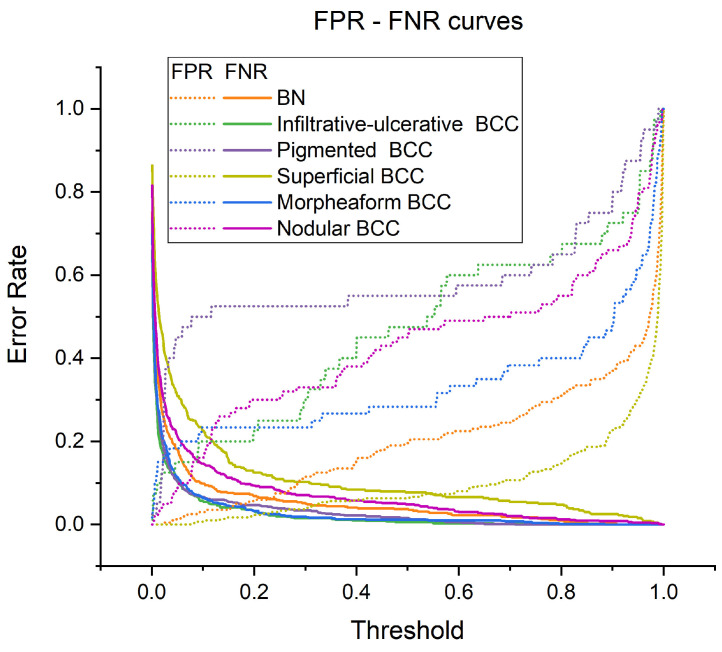
Dependence of the proportion of false positive and false negative predictions of the classifier on the established threshold for the probability of belonging to each diagnosis.

Figure 7 shows the dependences of the proportion of false negative results (FNRs) and the proportion of false positive results (FPRs) on the probability threshold at which the diagnosis is considered positive. In the model, these probability thresholds can be set to reduce the proportion of attribution of BCC to the BN group and/or the most aggressive clinical forms of BCC to the less aggressive group. When adjusting the threshold values, the number of false positive and false negative results increases or decreases, respectively, as shown in Figure 7. The rate of change in FPRs and FNRs is different for various clinical forms of BCC and BN. It can be observed, for example, that as the probability threshold increases, the proportion of FPRs slowly increases for BN and superficial BCC, while, for other forms of BCC, FPRs increases faster. The proportion of false negative predictions decreases very sharply.

**Figure 8:**
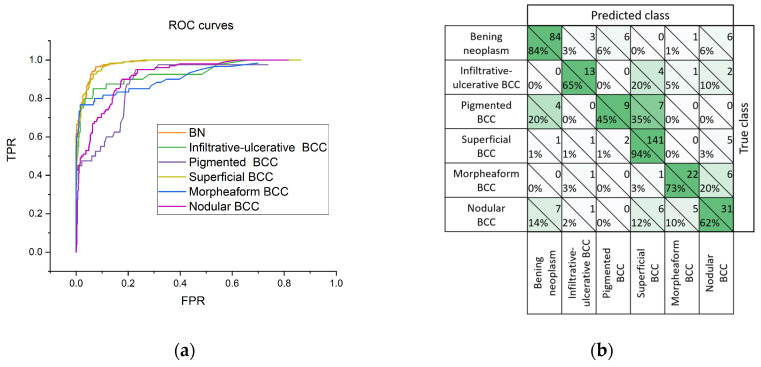
ROC curves (**a**) and confusion matrix (**b**) for various classes; in each cell of the adjacency matrix, the number of observations is indicated at the top right, and the proportion of observations of the true class that were assigned to the predicted class is indicated at the bottom left.

Figure 8 shows receiver operating characteristic (ROC) curves reflecting the dependence of the proportion of TPRs on FPRs, and the adjacency matrix, for the construction of which the model has produced a diagnosis corresponding to the highest value in the probability vector. According to the ROC curve (Figure 8a), the greatest diagnostic potential of the proposed classifier can be noted for BN and the superficial BCC. In Figure 8b, it can be seen that pigmented BCC in 35% of cases is classified as superficial BCC, which may be due to the absence or low sensitivity of OCT and US imaging methods for melanin pigmentation.

**Figure 9:**
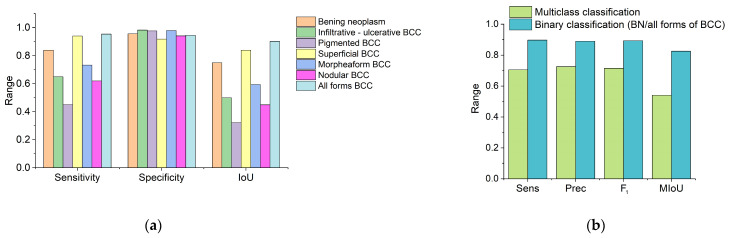
Histograms of sensitivity, specificity and IoU (**a**) sens, prec, F_1_ and mIoU (**b**) for the proposed classification method.

Figure 9 shows classification quality scores calculated from the adjacency matrix. High specificity can be noted for all classes of neoplasms. The greatest sensitivity of the proposed method for differentiating neoplasms is observed in the groups of BN and superficial BCC (Figure 9a). The highest value of the IoU corresponds to the BN, superficial BCC groups and for all forms of BCC in cases where the clinical form of BCC was not taken into account (Figure 9a). The average sensitivity and specificity of the proposed method for BN and various clinical forms of BCC were 70.6 ± 17.3% and 95.9 ± 2.5%, respectively. Figure 9b shows a histogram of the sensitivity, precision and F_1_ of the proposed algorithm for multiclass classification, which took into account the type of neoplasm and the clinical form of BCC, and binary classification, in which neoplasms were classified as benign or BCC without taking into account the clinical form. Sensitivity, precision, F_1_ and mIoU for multiclass classification were 70.6 ± 17.3%, 72.6 ± 14.2%, 71.5 ± 15.6% and 57.6% ± 20.1%, respectively, and, for binary classification, were 89.1 ± 8.0%, 89.0 ± 2.1%, 89.3 ± 3.4% and 82.6% ± 10.8%, respectively.

**Figure 10:**
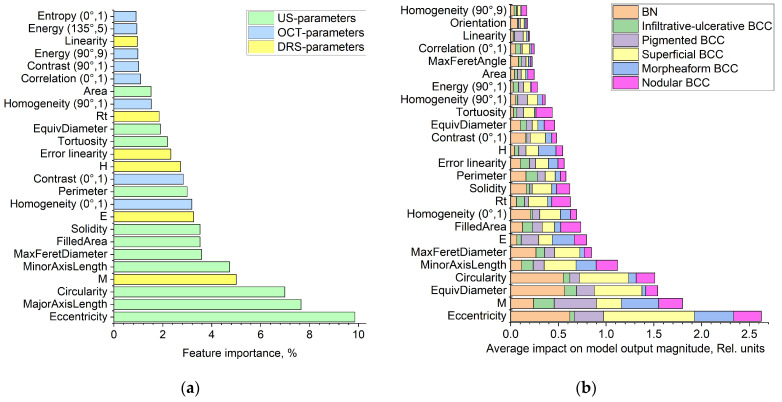
Feature importance histograms calculated using CatBoost (**a**) and SHAP (**b**).

Figure 10 shows histograms of the 25 most significant features that make the greatest contribution to the prediction of the proposed classifier, calculated using the built-in CatBoost functionality and the SHAP library. It is easy to notice that the first three most important parameters are the characteristics of the form of the neoplasm, and the first 25 parameters include parameters of all three modalities (Figure 10a). In addition, Figure 10b shows that the contribution of various features is different for all classes of tumors. For example, the Eccentricity parameter is one of the most important parameters in predicting the superficial clinical form of BCC, indicating that the morphological difference in the tumor shape from a perfect circle plays an important role in the classification of this type of neoplasm, while, for BN, the parameters of Eccentricity, EquivDiameter and Circularity have approximately the same value, which means that, for this type of tumor, the size and shape of the tumor have equal importance and influence on the classification. For the nodular clinical form of BCC, the most important parameters are eccentricity, melanin pigmentation coefficient—M, circulation, etc. For the pigmented type of BCC, the melanin pigmentation coefficient has the greatest contribution to predicting this diagnosis. In this article, we do not compare or correlate specific histopathological markers of each clinical neoplasm with the measured parameters.

## 4. Discussion

This paper proposes a method for differentiating skin neoplasms using a multimodal method and machine learning. The main differences in this approach from previously proposed ones are the ability to differentiate various clinical forms of BCC and the use of three screening methods: DRS, OCT and US, which are used as diagnostic tools.

The proposed characteristic parameters have shown viable diagnostic potential. The algorithm for classifying tumor types has given an average sensitivity and specificity of 70.6% and 95.9%. At the same time, for the BN class, the sensitivity and specificity are 84.0% and 95.6%, and, for the BCC class, without division into clinical forms of the tumor, they are 95.4% and 94.6%, respectively. The lowest sensitivity is observed in the pigmented and infiltrative–ulcerative BCC, which may be due to the small sample, insensitivity of OCT and US to melanin pigmentation and the presence of a hard crust over the neoplasm for the infiltrative–ulcerative BCC, which distorts OCT and US visualization and limits the effective probing depth.

The results highlight the potential of combined modality approaches, which show promise in overcoming the limitations inherent to individual imaging or spectroscopy techniques. While high-resolution OCT provides intricate structural details of skin morphology, US imaging offers insights into BCC depth and structure, and DRS adds functional tissue information, such as hemoglobin and melanin concentrations. Each modality’s limitations, when used in isolation, are mitigated by the complementary capabilities of the others.

In this work, we have not taken into account such parameters as the observed thickening of the epidermis, changes in the contrast between the epidermis/dermis boundary, increase in the number and size of optical inhomogeneities associated with blood and lymphatic microvessels in comparison with the control nearby healthy skin area, contrast of boundaries of visualized neoplasms and other quantitative or categorical characteristics. Accounting for such parameters can help to increase the diagnostic sensitivity and specificity of the proposed approach to differentiating neoplasms by type and clinical form.

A number of studies have shown an increase in sensitivity and specificity when using a combination of various diagnostic methods and machine learning. In particular, Ulrich et al. [44] noted an increase in the accuracy and specificity of diagnosing non-pigmented BCC with a combination of dermatoscopy and OCT. Accuracy and specificity increased from 76.2% and 54.3% for clinical examination/dermatoscopy to 87.4% and 75.3% for clinical examination/dermatoscopy/OCT, respectively. In this study, OCT images were assessed by a naked eye physician experienced in interpreting OCT scans for features affecting the epidermis and dermis. Sawyer et al. [39] used texture analysis of OCT images to diagnose ovarian tissue lesions using a quantitative assessment of the state of biological tissues. This approach allowed one to achieve an average classification accuracy of 78.6%. Marvdashti et al. [19] used texture analysis in addition to other OCT quantification methods for automated classification of BCC and healthy skin based on machine learning. According to the authors, this approach provided 95.4% and 95.4% sensitivity and specificity for the classification of BCC and healthy skin. Adabi et al. [20] used various texture analysis methods to extract features from OCT images in order to differentiate various skin neoplasms. The sensitivity and specificity of the developed method were 87.0% and 87.3%, respectively. Raupov et al. [45] developed a method for differentiating melanoma and BCC from benign nevus. Silver et al. [28,29] used vibration OCT to differentiate BCC, squamous cell carcinoma and melanoma from normal skin. This method demonstrated sensitivity from 83.3% to 91.6% and specificity from 77.8% to 87.5%. However, these studies did not examine the possibility of differentiating different clinical forms of BCC.

Longo et al. [46] proposed to use qualitative features for classifying different clinical forms of BCC using dermatoscopy and confocal microscopy images. This approach, which included 12 parameters, correctly classified 74.4% of the original cases.

The authors of refs. [21,22,23,24] used high-frequency US as a tool for the preoperative assessment and differentiation of various clinical forms of BCC according to the depth of invasion. The main parameters assessed were the size and depth of tumor invasion. Barcaui et al. [24] noted that the use of high-frequency US alone was not enough to confirm the diagnosis, but allowed for a detailed preoperative study by assessing the different layers of the skin and their corresponding thickness, assessing the size of the tumor and assessing the damage to the deep layers.

Refs. [25,26,27] showed the sensitivity of the DRS method for differentiating various neoplasms and conditionally healthy skin. Authors noted that the main spectral features during DRS screening were associated with the effects of the reabsorption of various forms of hemoglobin and melanin, as well as their concentrations in various pathologies. Garcia-Uribe et al. used an artificial neural network to differentiate BCC and squamous cell carcinoma from nonmelanoma skin lesions using DRS [47]. According to the authors, the classifier achieved 92% sensitivity and specificity.

Subjective perceptions of oncologists can lead to errors in diagnosis. In addition, the likelihood of a physician’s errors may depend on his/her relatively limited clinical experience, which potentially impacts patient outcomes and quality of care. The proposed approach to differentiating clinical forms of BCC can serve as the basis for the development of tools to support objective clinical decision making. It is worth noting that the development of methods and protocols for standardizing measured data is necessary to ensure consistency of results between different models of identical devices (for example, between different OCT models), which will help to maintain the reproducibility and accuracy of measurements in different conditions and eliminate the influence of technical features of the device model used.

Correct differentiation of various clinical forms of BCC can have significant clinical implications when choosing treatment tactics [13,48]. The proposed method may be a useful tool in deciding and justifying the extent of surgical interventions or considering alternative treatment modalities, such as topical treatment or photodynamic therapy, which are suitable for less aggressive clinical forms of BCC, such as superficial [13,48,49], while infiltrative or morpheaform BCC requires a more radical initial surgical approach compared to other subtypes [49].

Differentiation of BCC subtypes at the initial stage can help to assess the risk of relapse characteristic of each clinical form of the neoplasm. Thus, methods for non-invasive differentiation of the clinical form of BCC can be used to assess the risk of relapse and provide additional information necessary to justify the choice of treatment tactics and the urgency and volume of therapeutic interventions, as well as to select and prescribe an additional type of examination; for example: observation, incisional or excisional, or punch biopsy.

Despite the obvious imbalance in the class distribution, our model showed relatively good results in terms of F_1_ score and ROC curve, as well as accuracy and specificity, compared to previously proposed diagnostic methods. We believe that a larger sample and a more balanced dataset will improve estimates of the algorithm’s predictive ability. The main drawback of the proposed approach is associated with the spatial inconsistency of the measurements of DRS, OCT and US. The data of each modality were obtained from different areas of the tumor and compared with each other randomly. Thus, we may have lost or rendered insignificant cross-modal correlations of different parameters.

The proposed method requires external validation on independent datasets. We plan to perform this verification in the future.

Expanding our dataset is critical to improving diagnostic accuracy. This study is a pilot study. We are actively collaborating with healthcare institutions to gather more data that will not only enhance the robustness of our proposed method but will also allow for continuous performance improvement.

We believe that spatial consistency of DRS, OCT and US measurements will allow us to fully take into account and establish cross-modal correlations of various parameters and increase the diagnostic accuracy of the method. Spatial consistency of DRS, OCT and US measurements can be achieved by integrating all three screening methods into a single measurement probe. In the works [50,51], the combination of OCT and US into one measuring probe is described, allowing for simultaneous OCT and ultrasound visualization of the same area. As an alternative method for achieving spatial consistency of measurements, optical and acoustic markers can be developed that can be applied to the biological tissue under study to mark the same area, which will allow the results of measurements from different modalities to be consistent at the screening or post-processing stage.

In the future, we plan to improve model performance by fine-tuning the characteristic parameters and eliminating imbalanced datasets through more comprehensive preprocessing, including optimization of the tumor screening protocol and larger sample sizes, which will make the sample more balanced. Further improvements can be achieved by using additional quantitative or categorical features and removing unnecessary features that do not contribute to the predictive ability of the proposed classification algorithm. We also plan to pay more attention to the features that are most useful for correct classification. We hope that this approach will reduce the computational cost. We believe that the proposed method will be useful for the early detection of disease relapse. We are currently collecting data on the use of the proposed method to detect relapse on several volunteers. The results of this study will be published later.

In addition, future use of less stringent clinical classification may result in higher diagnostic rates. For example, a diagnostic algorithm that categorizes BCCs into broader categories based on their clinical behavior and aggressiveness, rather than focusing solely on histopathological variations, may be simpler and more useful in clinical practice.

Although the focus of our research is on the analysis and differentiation of different clinical forms of BCC, the basic principles of our method may be applicable to a wider range of skin diseases. We hypothesize that this method can be adapted or extended to analyze other skin diseases, including differentiating other types of cancer and their different clinical forms, including melanoma. Testing of the developed method on other types of cancer or skin conditions was beyond the scope of this study; however, it represents an important direction for future research.

## 5. Conclusions

We have developed a method for differentiating benign neoplasms and various clinical forms of BCC based on a multimodal diagnostic method and machine learning. The diagnostic potential of the multimodal method for studying skin tumors is noted. This is the first study that is based on the simultaneous use of DRS, OCT and high-frequency US for the differentiation of skin neoplasms. The proposed classification algorithm has demonstrated an average sensitivity of 70.6 ± 17.3%, specificity of 95.9 ± 2.5%, precision of 72.6 ± 14.2%, *F*_1_-score of 71.5 ± 15.6% and mIoU of 57.6 ± 20.1% for diagnostics of the main clinical form of BCC. Without taking into account the clinical form of the tumor (i.e., BN vs. all forms of BCC), values of sensitivity/specificity/precision/*F*_1_-score/mIoU significantly increased and achieved 89.1 ± 8.0%/95.1 ± 0.7%/89.0 ± 2.1%/89.3 ± 3.4%/82.6 ± 10.8%, respectively.

It is important to note that, from the point of view of introducing the proposed methods into clinical research practice, the equipment used is standard and commercially available. Its use does not require special training of personnel, and the processing of measurement results can be automated over time.

Integrating DRS, OCT and US findings holds the potential to reduce false positives and negatives, thereby enhancing diagnostic confidence for various forms of BCC and BN. Although the multiclass classification achieved favorable accuracies in differentiating neoplasms when considered en masse, the approach still requires further development to reliably discern between the more nuanced presentations of individual BCC types.

The results also emphasize the utility of machine learning as a tool for augmenting diagnostic precision, a notable stride for objective clinical decision making. However, augmenting the classifier’s performance hinges on addressing the current imbalances in data and incorporating additional clinically relevant parameters. Additionally, standardization of data across different modalities and equipment models remains a crucial step for broad applicability in diverse clinical settings.

Moving forward, an expansion of the dataset and continued refinement of the classifier will likely yield more consistent and robust diagnostic outcomes. Subsequent validation on larger, independent datasets will be indispensable in affirming the method’s reliability. Lastly, this research paves the way for future exploration of integrated diagnostic tools in dermatological oncology, with broader implications for a range of skin diseases well beyond BCC and BN. Such advancements are anticipated to enrich the clinical decision-making process, contributing significantly to patient care and management strategies in oncological dermatology.

## Data Availability

Data available on request from the corresponding author due to confidentiality restrictions.
